# Combination of specific allergen and probiotics induces specific regulatory B cells and enhances specific immunotherapy effect on allergic rhinitis

**DOI:** 10.18632/oncotarget.10946

**Published:** 2016-07-29

**Authors:** Ling-Zhi Xu, Li-Tao Yang, Shu-Qi Qiu, Gui Yang, Xiang-Qian Luo, Bei-Ping Miao, Xiao-Rui Geng, Zhi-Qiang Liu, Jun Liu, Zhong Wen, Shuai Wang, Huan-Ping Zhang, Jing Li, Zhi-Gang Liu, Hua-Bin Li, Ping-Chang Yang

**Affiliations:** ^1^ ENT Institute of The Research Center of Allergy & Immunology, Shenzhen University School of Medicine, Shenzhen, China; ^2^ Longgang ENT Hospital, Shenzhen, China; ^3^ Department of Otolaryngology, Head and Neck Surgery, Xinhua Hospital, Shanghai Jiaotong University School of Medicine, Shanghai, China; ^4^ Department of Otolaryngology, The First Affiliated Hospital of Shenzhen University, Shenzhen, China; ^5^ Shenzhen Maternity & Child Health Hospital, Shenzhen, China; ^6^ Department of Otolaryngology of Zhujiang Hospital, Southern Medical University, Guangzhou, China; ^7^ Brain Body Institute, McMaster University, Hamilton, ON, Canada; ^8^ Department of Allergy and Clinical Immunology, State Key Laboratory of Respiratory Disease, The First Affiliated Hospital, Guangzhou Medical University, Guangzhou, China

**Keywords:** allergic rhinitis, specific immunotherapy, probiotics, regulatory B cells, immunoglobulin E, Immunology and Microbiology Section, Immune response, Immunity

## Abstract

The therapeutic efficacy of allergen specific immunotherapy (SIT) on allergic diseases is to be improved. Probiotics can regulate immune response. This study aims to promote the effect of SIT on allergic rhinitis (AR) by co-administration with *Clostridium butyricum* (Cb). In this study, patients with AR sensitized to mite allergens were enrolled to this study, and treated with SIT or/and Cb. The therapeutic efficacy was evaluated by the total nasal symptom scores (NSS), medication scores, serum specific IgE levels and T helper (Th)2 cytokine levels. The improvement of immune regulation in the AR patients was assessed by immunologic approaches. The results showed that treating AR patients with SIT alone markedly reduced NSS and medication scores; but did not alter the serum specific IgE, Th2 cytokines and skin prick test (SPT) index. The clinical symptoms on AR in SIT group relapsed one month after stopping SIT. Co-administration of Cb significantly enhanced the efficacy of SIT on AR as shown by suppression of NSS, medication scores, serum specific IgE, Th2 cytokines and SPT index; the regulatory B cell frequency was also markedly increased. Such an effect on AR was maintained throughout the observation period even after stopping the treatment. Butyrate blocked the activation of histone deacetylase-1, the downstream activities of epsilon chain promoter activation, and the IgE production in the antigen specific B cells. On the other hand, butyrate induced the IL-10 expression in B cells with a premise of the B cell receptor activation by specific antigens. In conclusion, administration with Cb can markedly enhance the efficacy of SIT on AR.

## INTRODUCTION

The allergen specific immunotherapy (SIT) is extensively employed as a specific therapy for allergic diseases [1]. The studies of SIT are advancing rapidly in both clinic and laboratory in the recent decades. However, although the therapeutic efficacy of SIT on allergic diseases is recognized, its efficacy still needs to be further improved [2] and the underlying mechanism is to be further elucidated.

Allergic rhinitis (AR) is a disease mainly mediated by antigen specific immunoglobulin (Ig)E [3]. Upon re-exposure to specific antigens, the antigen specific B cells differentiate into plasma cells to produce antigen specific IgE; the latter binds to the high affinity IgE receptors on the surface of mast cells to make the mast cells sensitized. Upon re-exposure to specific antigens, the sensitized mast cells degranulate and release allergic mediators to initiate allergic symptoms [4]. A key change is induced in the antigen specific B cells in response to re-exposure to specific antigens; the antigens trigger the IgE class-switch and the gene of the Ig ε heavy chain transcription, to render the antigen specific B cells to differentiate into plasma cells to produce specific IgE [5]. The underlying mechanism is unclear; how to modulate the process of the Ig ε chain expression in B cells is unknown.

SIT is a therapeutic method by introducing in small doses of specific antigens to patients via subcutaneous injection or sublingual absorption. The current understanding of SIT is to restore tolerance to the allergen by reducing its tendency to induce IgE production. It is reported that SIT promotes the generation of regulatory T cells (Treg) [6] and regulatory B cells (Breg) [7], which is explicated playing an important role in the SIT's efficacy in suppressing allergic symptoms. However, the mechanism by which SIT modulates the generation of immune regulatory cells under an allergic environment is far from clear. On the other hand, the efficacy of SIT in the treatment of allergic diseases varies greatly in different reports. Some investigators found that SIT resulted in the greatest improvement in allergic symptoms [8–10] while others noted less effect of SIT on allergic diseases [11–13]. The underlying mechanism is unclear.

Probiotics, such as *Lactobacillus* and *Bifidobacterium*, are the good bacteria in the human intestinal tract and have been used to improve health conditions [14, 15]. Unfortunately, the direct connection between taking these products and improving immune function has not yet been elucidated. Our previous studies observed that, *Clostridium butyricum* (Cb), one of the probiotics, could attenuate the airway allergic inflammation via improving the nasal epithelial barrier function [16]. It is also demonstrated that butyrate, one of the products of Cb, can induce antigen-specific anergy in both cloned and naïve CD4^+^ T cells [17]. Therefore, we hypothesize that administration with Cb may enhance the therapeutic efficacy of SIT. In this study, we treated AR patients with SIT and Cb. The results showed that the administration with Cb significantly enhanced the therapeutic efficacy of SIT on AR symptoms. The presence of butyrate inhibited the Ig ε gene transcription, promoted the expression of IL-10 by antigen specific B cells, and thus facilitated the generation of Bregs.

## RESULTS

### Administration with Cb enhances the efficacy of SIT on allergic rhinitis (AR)

**Figure 1:**
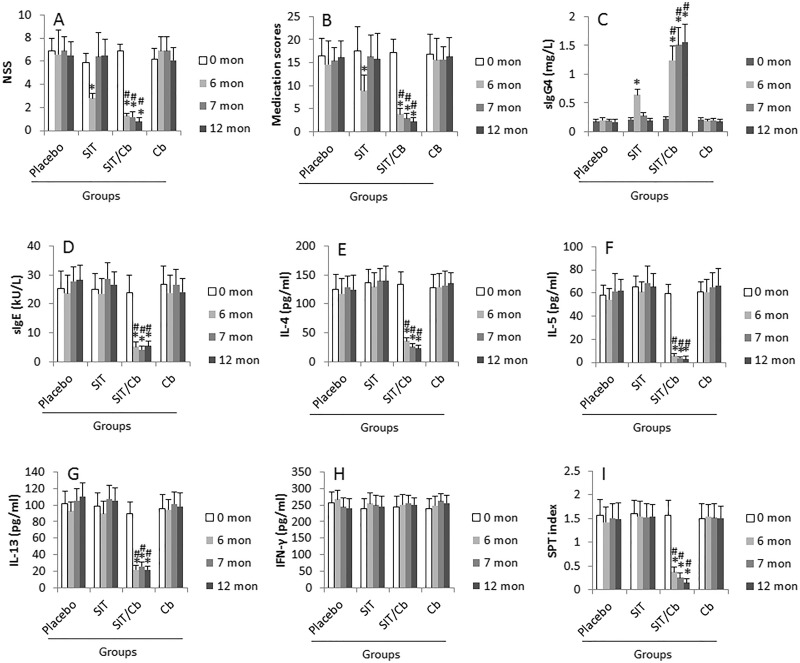
Assessment of AR status AR patients were treated as denoted on the X axis of the bar graphs. Placebo: Patients were treated with placebo (*n* = 20), or SIT (*n* = 46; group1), or SIT/Cb (Cb = *C*. *butyric*; *n* = 44; group2), or Cb (*n* = 48) respectively. **A.** The NSS were recorded once a week. The bars indicate the averaged NSS of 2 weeks prior to the indicated time-points. **B.** The bars indicate the medical scores of 2 weeks prior to the indicated time-points. **C.**-**H.** The sera were collected from each patient at month 0, 6, 7 and 12 respectively and analyzed by ELISA. **I.** The bars indicate the SPT results at the indicated time-points. Data of bars are presented as mean ± SD. *, *p* < 0.01, compared to the placebo group. #, *p* < 0.01, compared with the SIT group.

The AR patients were treated with SIT or/and Cb, or placebo. The NSS and medication scores from each patient were recorded once a week. The sera were collected from each patient in months 0, 6, 7 and 12 respectively. All the participants completed the study. As compared with the placebo group, treatment with SIT for 6 months markedly improved the NSS (Figure 1A) and medication scores (Figure 1B, Table 2), increased serum specific IgG4 (Figure 1C); but did not apparently down regulate the specific IgE (Figure 1D), Th2 cytokines (Figure 1E-1H) in the serum and the SPT index (Figure 1I, which were further improved in patients treated with both SIT/Cb. From month 7 to 12, all the patients were treated with placebo. The data from month 7 showed a great difference between the SIT/Cb group and the SIT group. In the SIT group, the NSS, medication scores at month 7 almost returned to the levels of month 0, while these parameters were maintained at the levels of month 6 and lasted throughout the observation period in the SIT/Cb group. We also assessed the serum level of IFN-γ during the treatment; not much changes were induced by the treatment with SIT or/and Cb (Figure 1H). The results demonstrate that treating AR patients with SIT for 6 months can attenuate about 1/3 clinical symptoms based on the NSS, but the AR clinical symptoms return to the levels of before treatment within one month, which can be prevented by treating with SIT/Cb. As analyzed by correlation assay, the serum specific IgE levels were positively correlated with the SPT index (*r* = 0.618; *p* < 0.01). In addition, no serious local or systemic adverse reaction of SIT was observed in this study.

**Table 1:** Characteristics of AR patients

	Placebo	SIT	SIT/Cb	Cb
Number of Subjects	20	46	44	48
Age (yr) (median)	26	25	25	26
Male Female	10 (50) 10 (50)	22 (47.8) 24 (52.2)	21 (47.7) 23 (52.3)	25 (52.1) 23 (47.9)
SPT (diameter)* <3 mm 10-15 mm >15 mm	0 8 (40) 12 (60)	0 18 (39.1) 28 (61)	0 17 (38.6) 27 (61.2)	0 19 (39.6) 29 (60.4)
HDM sIgE^#^ 17.5-50 KU/L 50-100 KU/L >100 KU/L	2 (10) 13 (65) 5 (25)	5 (10.9) 28 (60.9) 13 ((28.3)	5 (11.4) 27 (61.4) 12 (27.3)	5 (10.4) 28 (58.3) 15 (31.3)

* A wheal diameter greater than 3 mm of the negative saline control was considered SPT positive.

# The serum sIgE to HDM (*Der p* and *Der p*) was measured by the ImmunoCap test and a value of more than 0.35 kUA/L was considered a positive response.

**Table 2:** Medication scores^$^

	Placebo	SIT	SIT/CB	CB
Month 0^#^	16.5 ± 3.8	17.6 ± 5.2	17.1 ± 2.9	16.8 ± 4.4
Month 6	14.6 ± 5.2	8.9 ± 3.4*	3.8 ± 1.2*	15.6 ± 4.6
Month 7	15.4 ± 2.9	16.3 ± 4.7	2.9 ± 1.1*	15.7 ± 2.8
Month 12	16.1 ± 3.6	15.8 ± 5.5	2.2 ± 0.8*	16.3 ± 4.1

$ The data are an average of medication score per week.

# medication scores at month 0 is the scores of two weeks before treatment.

* *p* < 0.01.

### SIT/Cb therapy promotes the generation of regulatory B cells (Bregs) in AR patients

**Figure 2:**
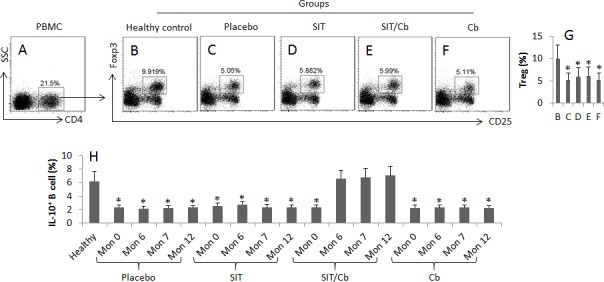
SIT/Cb promotes generation of Bregs in AR patients The number of patient and the patient information is the same as Figure 1. The peripheral mononuclear cells (PBMC) were collected from each patient and analyzed by flow cytometry. **A.** The gated cells show the CD4^+^ T cells in LPMCs. **B.**-**F.** The gated cells show Tregs in the CD4^+^ T cells of panel A. **G.** The bars indicate the summarized data of B-F. **H.** The bars indicate the frequency of IL-10^+^ B cells in PBMCs. The data of bars are presented as mean ± SD. *, *p* < 0.01, compared with group B (G), or the healthy group (H). Samples from individual patients were processed separately. Cb: *C. butyricum*.

After the treatment with SIT or/and Cb, the frequencies of the peripheral Tregs and Bregs were evaluated by flow cytometry with blood samples from AR patients. The results showed that, after treatment for 6 months, the frequency of peripheral Tregs in the group treated with placebo was significantly lower than healthy controls. Treatment of AR patients with either SIT or/and Cb only slightly up regulated the frequency of Tregs (p>0.05. Figure 2A-2G).

As shown by Figure 2H, the frequency of peripheral Bregs was lower in AR group than the healthy subjects. After treatment for 6 months, the frequency of Breg was up regulated in the SIT/Cb group, which was maintained throughout the observation period. The frequency of Bregs was not apparently altered in either SIT group, or Cb group, or placebo group.

### Butyrate and Der p 1 modulates antigen specific B cell differentiation

The data of Figure 1 and Figure 2 suggest that administration of SIT/Cb promotes the generation of Bregs. To assess the antigen specific B cell in the peripheral system, we isolated CD19^+^ B cells from AR patients and cultured in the presence of Der p 1 (specific antigen; verified by ourselves as demonstrated by Figure S1 and Table S1 in supplemental materials) or BSA (an irrelevant antigen) for 3 days. As shown by flow cytometry assay, exposure to Der p 1, but not BSA, induced marked proliferation of B cells (Figure S2). The data indicate that these proliferating B cells are Der p 1-specific B cells.

**Figure 3:**
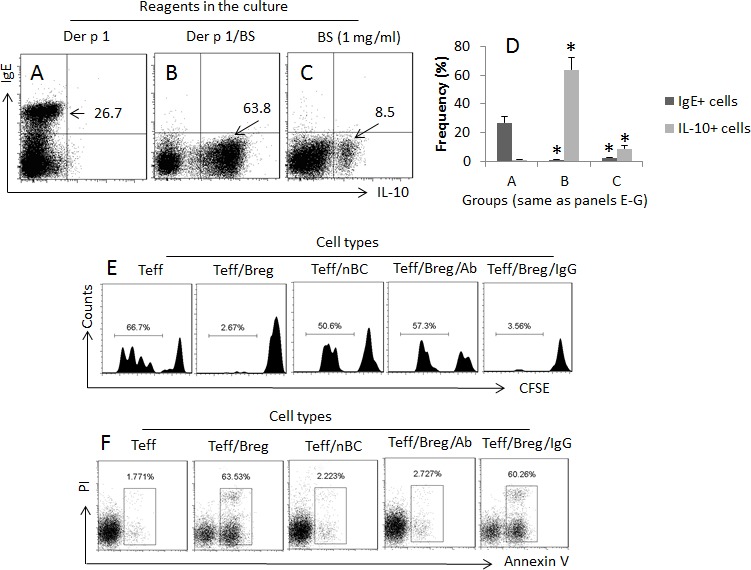
Specific antigens and butyrate differentially modulate antigen specific B cell properties DerpsBCs were isolated from 12 AR patients (samples from each patient were used for one test). **A.**-**D.** The dot plots show the frequencies of IgE or/and IL-10 positive B cells in response to the treatment (denoted above each panel; anti-CD40 was added to the culture at 20 ng/ml). BS: Butyrate sodium (1 mg/ml). The bars indicate the summarized data of A-D. **E.** The histograms indicate Teff cell proliferation, **F.** The gated dot plots indicate the frequency of apoptotic Teff cells, in response to the treatment (denoted above each panel). Teff: Breg (or naive B cell; nBC) = 10^4^:10^4^/ml. Teff cells were activated by the presence of anti-CD3 (2 μg/ml)/CD28 (5 μg/ml). Bregs were activated by the specific antigen, Der p 1 (1 μg/ml). Ab: Anti-IL-10 (1 μg/ml). IgG: Isotype IgG (1 μg/ml; control Ab).

We then observed the different effects of administration of SIT or/and Cb on the differentiation of Der p 1-specific B cells. The peripheral Der p 1-specific B cells (DerpsBCs) were isolated from AR patients before any specific treatment and cultured in the presence of Der p 1 or and Cb for 4 days. As analyzed by flow cytometry, about 26.7% DerpsBCs showed IgE positive; about 63.8% became IL-10^+^ B cells in response to Derp1/Cb stimulation, while only 8.5% IL-10^+^ B cells were detected in the presence of Cb (Figure 3A-3D). The IL-10^+^ Bregs showed an immune suppressor ability on CD4^+^ effector T cell proliferation (Figure 3E) and induction of T cell apoptosis (Figure 3F). The results indicate that exposure to Der p 1 alone induces DerpsBCs to differentiate into IgE-producing cells. Exposure to both Der p 1 and butyrate induces DerpsBCs to differentiate into IL-10-producing cells.

### The cooperative effect of B cell receptor activation and butyrate on differential modulation of IgE and IL-10 expression in DerpsBCs

**Figure 4:**
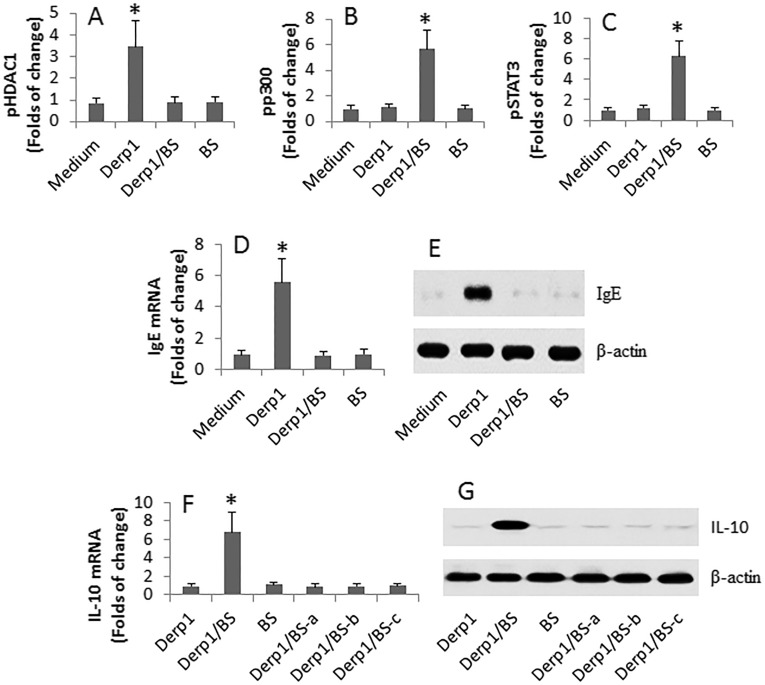
Exposure to Der p 1 and butyrate modulates IgE expression in DerpsBCs DerpsBCs were isolated from 30 AR patients. The cells were stimulated with Der p 1 or/and BS in the culture for 48 h. The cells were collected and analyzed by ChIP, RT-qPCR and Western blotting. **A.**-**C.** The bars indicate the pHDAC1 (A), pp300 (B) and pSTAT3 (C) at the IgE promoter locus. **D.**-**E.** The bars indicate the mRNA levels and the immune blots show the protein levels of IgE in the DerpsBCs. **F.**-**G.** the bars indicate the mRNA levels and the immune blots show the protein levels of IL-10 in the DerpsBCs. a, b, c: Btk (a; PCI-32765 = 1 mM), p300 inhibitor (b; C646; 1 mM) and STAT3 inhibitor (c; SC-1; 15 mM) were added to the culture. Der p 1: 1 μg/ml. BS: 1 mg/ml. *, *p* < 0.01, compared with the medium group.

Since butyrate is an inhibitor of the histone deacetylase (HDAC)1 [16], the data of Figure 3 suggest that HDAC1 may play a role in the DerpsBC differentiation. To this end, we analyzed the phosphorylation status of HDAC1 in DerpsBCs after treatment with Der p 1 or/and butyrate in the culture. The results showed that exposure to Der p 1 markedly increased the levels of pHDAC1 at the Igε chain gene promoter locus of the DerpsBCs, which did not occur in those treated with both Der p 1 and butyrate, or in those treated with butyrate alone (Figure 4A). In line with published data that p300 and STAT3 are involved in the expression of IL-10 [18], we also detected marked enhancement of the pp300 and pSTAT3 levels at the IL-10 promoter locus in DerpsBCs after exposure to both Der p 1 and butyrate in the culture, but not in exposure to either Der p 1 alone or butyrate alone (Figure 4B, 4C). Further experiments showed that the levels of IgE were markedly increased in DerpsBCs (Figure 4D, 4E). In addition, exposure to Der p 1/butyrate (but not either Der p 1 or butyrate alone) significantly increased the expression of IL-10 in the DerpsBCs (Figure 4F, 4G), which was abolished in the presence of the BCR signal inhibitor, or p300 inhibitor, or STAT3 inhibitor, in the culture.

## DISCUSSION

Similar to published data [8, 11, 19], the present study shows that treatment with SIT for 6 months attenuated about 52.5% of the clinical symptoms of AR. Co-administration with SIT and Cb significantly enforced the therapeutic effect on AR (attenuated 81.2% of the AR symptoms). Therefore, the data indicate that administration with Cb markedly potentiates the therapeutic efficacy of SIT on the clinical symptoms of AR patients. Supportive results were also observed by others. Such as Kaufman did a 2-year SIT for allergic dermatitis, up to 81% symptom improvement was observed [9]. Warner et al noted “the greatest improvement in asthma symptoms” after treatment with SIT [10].

To test the efficacy of SIT on suppressing AR symptoms, we treated AR patients with SIT for 6 months first, then treated with placebo for the subsequent 6 months. However, the AR symptoms returned to the levels before SIT within one month. Such a result may be explained by the basic immunological knowledge. We only introduced in small doses of the specific allergens to AR patients in SIT. The specific antigens inevitably bind to and activate the sensitized mast cells. In this way, the allergic mediators in the mast cells may be slowly released and exhausted. But the mast cells are still alive, and are potentially to be activated once SIT is stopped. The inference is supported by the subsequent data. When SIT was stopped for one month, the AR symptoms relapsed in all the AR patients in the SIT group.

The present data show that the major immunological parameters, including the specific IgE and Th2 cytokines, were not apparently altered in the AR patients after treatment with SIT for 6 months, suggesting that SIT alone may be not able to modulate the skewed Th2 polarization status and the highly specific IgE production in AR patients. Other investigators also observed similar phenomenons. Such as Roger et al reported that no significant changes were observed in concentrations of total IgE, specific IgE or cytokines (IFN-γ, IL-4, IL-5, IL-10 and IL-13) in AR patients after SIT although satisfactory relief of AR symptoms was declared by most patients in the period of the treatment [11]. Glover et al indicated that the efficacy of SIT on asthma was “uncertain” [12] while Galli even observed no difference in the therapeutic efficacy between the SIT group and the control group [13].

It is suggested that probiotics can improve immune function in the body [20]. The effect of probiotics on allergic diseases has not been determined yet [21]. By reviewing literature, overall data is conflicting and evidence is limited [22]. Moderate effect on prevention of allergic dermatitis is reported, but not in AR or asthma [23, 24]. Our data are in line with those reports. Treating AR patients with Cb alone did not show an apparent suppressing effect on AR clinical symptoms or on the levels of specific IgE and Th2 cytokines in the sera. The significant aspect revealed by the present study is that co-administration with Cb and SIT markedly potentiated the therapeutic effect on AR, as well as improved the immunological parameters, including down regulation of specific IgE and Th2 cytokines, and up regulated Breg generation in AR patients. It is noteworthy that the treatment with both SIT and Cb was 6 months; the inhibition of AR symptoms and the improvement of the immunological parameters were maintained in the patients at least for 12 months, even though the patients were treated with placebo in the period of 7-12 months.

Butyrate is one of the products of Cb; it is an inhibitor of histone deacetylases and has multiple functions. Our recent work showed that butyrate could improve the airway epithelial barrier function [16]. HDAC1 expression was increased in patients with severe asthma compared with healthy volunteers [25]. Our data show that the exposure to specific antigens activates HDAC1 in the DerpsBCs and induces these cells to differentiate into specific IgE producing plasma cells. This phenomenon supports the data that treating AR patients with SIT alone did not alter the skewed Th2 polarization status and the high production of specific IgE, while treating the patients with both SIT and Cb suppressed the production of IgE and increased the production of IL-10.

Both p300 and STAT3 are involved in the expression of IL-10 [18]. The present data also show that exposure to butyrate activated p300 and STAT3 in DerpsBCs and induced these cells to differentiate into IL-10-producing B cells. Since these cells showed the ability to suppress effector T cell proliferation and to induce T cell apoptosis, the cells may be designated as Bregs. Others also found that IL-10 inhibited IL-5 and GM-CSF expression that is also important action in the inhibition of allergic response [26]. The results also showed that exposure to butyrate alone did not induce IL-10 expression in B cells; the activation of B cell receptor was required. This point may be the reason that treatment with both SIT and Cb obtained satisfactory therapeutic effect on AR, while with either SIT alone or Cb alone did not.

Our data show that after treatment with SIT/Cb, the proportion of allergen-specific B cells was reduced while the proportion of Bregs increased in the circulation of AR patients. Our previous data also show that SIT can induce CD35^+^ B cells to improve the immune tolerance in the intestine [27]. Blair et al found that, after stimulation with CD40, the CD19^+^ CD24^hi^ CD38^hi^ B cells produced high levels of IL-10 and suppressed the differentiation of Th1 cells [28]. Iwada et al found that the CD24^hi^ CD27^+^ B cells produced IL-10 after a non-specific stimulation in the culture, from which the B cells gained the suppressor ability to inhibit cytokine production by monocytes [29]. Similar to our data, Stanic et al also found that the IL-10-overexpressing B cells suppressed IgE production as well as the proinflammatory cytokine production [30]. It seems that several subtypes of B cells can produce IL-10 to contribute to immune tolerance.

In summary, the present study showed that treating AR patients with Cb markedly enhanced the therapeutic efficacy of SIT.

## MATERIALS AND METHODS

### Study subject enrollment and ethic statement

The AR patients were enrolled into this study at the ENT Hospital of Shenzhen; the Department of Otolaryngology, Head & Neck Surgery, Shanghai Xinhua Hospital, Shanghai Jiaotong University School of Medicine; the Department of Otolaryngology, the First Affiliated Hospital of Shenzhen University and the Department of Otolaryngology, Zhujiang Hospital, Southern Medical University. The study was approved by the Human Ethic Committees at local universities. An informed written consent was obtained from each subject. The study was carried out in accordance with the approved guidelines.

### Study design

AR was diagnosed by our physicians based on the routine procedures of our hospitals as well as reported by others [31, 32]. The enrollment criteria include: they had Der p-specific IgE in the serum (assessed by ImmunoCap; Phadia, Uppsala, Sweden); (b) they had perennial AR for more than two years; (c) they did not have the history of apparent asthma; and (d) had not received allergen-specific immunotherapy; (e) they did not complicate chronic rhinosinusitis; (f) skin prick test (SPT; see the procedures in the supplemental materials) showed that they were only sensitive to house dust mite. The demographic data are presented in Table 1. The patients were randomly divided into 4 groups and treated with placebo, SIT, SIT/Cb and Cb, respectively. Six months later, the patients of both SIT group and SIT/Cb group were treated with placebo until the end of 12 months. The observation was completed in 12 months.

### SIT procedures and administration of Cb

With a well-established therapy [33], the SIT was performed in AR patients using the house dust mite extracts (Allergopharma Joachim Ganzer KG; Reinbek, Germany), or saline (placebo), via subcutaneous injection in 1 ml saline. The patients were coded. The observers were not aware of the code to avoid the observer bias. The SIT was initiated at a dosage of 20 U, and was continued weekly with an increase in the dosage each week; the dosages were 20, 40, 80, 200, 400, 800, 2,000, 4,000, 8,000, 10,000, 20,000, 40,000, 60,000, 80,000 and 100,000 U, respectively. The maintenance dose was 100,000 U once a week until the end of the 6^th^ month. The Cb was prescribed by physicians; the patients took two capsules of Cb (420 mg/capsule) or similar capsules containing placebo vehicle (Kexing Biotech, Shangdong, China. Cb is an over-the-counter clinical medicine [34]) twice daily.

### Nasal symptom score (NSS)

The severity of the nasal symptoms of the AR patients before and after treatment was assessed according to published procedures [35]. The global discomfort caused by AR was rated on a 0-10 scale, 0 being no symptom, and 10 being the maximal severity of the symptom. The NSS was recorded weekly.

### Medication scores

Following published procedures [31] with minor modifications, the medication scores were recorded for each patient during the period of treatment. When necessary, the patients were allowed to take anti-histamine tablets (levocetirizine; 5 mg/tablet) or/and nasal spray of corticosteroid (mometasone). 1 point: used one nasal spray per day; 2 points: used one tablet per day; 3 points, used one spray and one tablet per day. The results are presented as the averages per week.

### Derp1-probe construction

Following published procedures [36, 37], we constructed a specific Derp1-probe used to isolate Der p 1 specific B cells (DerpsBC). The Der p 1 was cloned and purified in our laboratory and labeled with biotin (data not shown). The biotinylated Der p 1 was incubated with magnetic particle-conjugated streptavidin for 30 min at room temperature. The unconjugated reagents were filtered through a filter tube by centrifugation. The Der p 1 covered magnetic beads were used for MACS isolation of DerpsBC.

### DerpsBC (Der p 1-specific B cell) isolation

Following published procedures [37], PBMCs were isolated from the peripheral blood, and incubated in the presence of Derp1-probe (2 μg/ml) for 30 min. The cells were then passed through the columns in the magnetic apparatus provided by Miltenyi Biotech. Cells were collected, washed with acidic phosphate-buffered saline (PBS) (pH 3) to remove the bound Der p 1 on the cell surface, and transferred to RPMI 1640 medium for further experiments.

### Chromatin IP (ChIP)

The cells were collected and fixed with 1% formaldehyde for 15 min to cross link the DNA and the bound proteins. The samples were sonicated to shear DNA along with bound proteins into small fragments of 300-500 bp. Cell lysate was precleared with incubating with protein G-agarose for 2 h at 4°C. The supernatant was incubated overnight at 4°C with 2 μg of specific antibodies or isotype IgG. The antibodies-DNA-protein complexes were isolated by centrifugation. After cross-link-reversal and DNA purification, qPCR was performed on the samples and inputs. The primers of the promoter regions of Ig heavy chain germline Igε include tgggcctgagagagaagaga and agctctgcctcagtgctttc. The IL-10 promoter primers are aactggctccccttaccttc and catggaggctggataggagg. The results are presented as relative changes against the input.

### Data analysis and statistics

Samples from individual patients were analyzed separately. Each experiment was repeated at least 3 times. Statistical analyses were performed using the Microsoft Excel. Symptom scores and serum measurements before and after immunotherapy were compared between two groups using the paired Student *t*-test or ANOVA if more than two groups. A correlation assay was performed with the data of serum specific IgE and the SPT index. A p value less than 0.05 was set as a significant criterion.

In addition, reagent information, procedures of skin prick test (SPT), ELISA (enzyme-linked immunosorbent assay), isolation of immune cells, cell culture, flow cytometry, assessment of apoptosis, real time quantitative RT-PCR (RT-qPCR), preparation of cytosolic and nuclear extracts and Western blotting are presented in the supplemental materials.

## SUPPLEMENTARY MATERIALS


