# Supplementary material for: Combination of specific allergen and probiotics induces specific regulatory B cells and enhances specific immunotherapy effect on allergic rhinitis
Source: Oncotarget. 2016 Jul 29;7(34):54360–9. doi: 10.18632/oncotarget.10946 (PMC5342347; doi:10.18632/oncotarget.10946)
Supplement: Supplementary file 1 [file oncotarget-07-54360-s001.pdf]

Combination of specific allergen and probiotics induces specific regulatory B cells and enhances specific immunotherapy effect on allergic rhinitis

Supplementary Material

Reagents

The antibodies against IgE, IL-10, HDAC1, pHDAC1, p300, pp300, STAT3, pSTAT3 were purchased from Santa Cruz Biotech (Santa Cruz, CA). The Fluorochrome-labeled antibodies of CD25, CD4, CD19, IL-10, Foxp3 were purchased from BD Biosciences (Franklin Lakes, NJ). The magnetic cell sorting kits were purchased from Miltenyi Biotech (San Diego, CA). The reagents for real time RT-PCR and the reagents for Western blotting were purchased from Invitrogen (Carlsbad, CA). The p300 inhibitor C646, STAT3 inhibitor SC-1, butyrate sodium, Annexin V kit, PCI-32765, protein G and the ChIP kit were purchased from Sigma Aldrich (St. Louis., MO). The ELISA kits of IgE, IL-4, IL-5, IL-13 and IFN-γ were purchased from R&D Systems (Minneapolis, MN). The biotinylated IgE antibody was from Abcam (Cambridge, MA). The capsules of Cb were produced by Kexing Biotech (Shangdong, China).

Skin prick test (SPT)

All patients were undergone a SPT with the following allergen extracts (ALK-Abelló, Hørsholm, Denmark): *D. pteronyssinus, D. farinae*, grass pollen mix, cat and dog dander, American cockroach, mould mix, tree pollen mix and weed pollens. The mean of the largest diameter of the wheal was recorded as the response to the SPT. Histamine (0.01 mg/ml) was used as a

positive control and saline was used as a negative control. The skin test index was the ratio between the response to an allergen and the response to histamine.

ELISA (enzyme-linked immunosorbent assay)

The sera were separated from the blood samples collected from each subject. The levels of DEM-specific IgE, DEM-specific IgG4, IL-4, IL-5, IL-13 and IFN-γ in the sera were determined by ELISA with commercial reagent kits following the manufacturers' instructions.

Isolation of immune cells

The peripheral blood was collected from AR patients and healthy volunteer subjects (40 ml/person). The peripheral mononuclear cells (PBMC) were isolated by the gradient density centrifugation. The immune cells were purified from PBMCs by the magnetic cell sorting (MACS) with commercial reagent kits following the manufacturer's instructions. The purity of the isolated cells was assessed by flow cytometry. If the purity did not reach 95%, MACS was performed again with the cells. The viability of the cells was greater than 98% as checked by Trypan blue exclusion assay.

Cell culture

The immune cells were cultured in RPMI1640 medium supplemented with 10% fetal bovine serum, 100 U/ml penicillin, 0.1 mg/ml streptomycin, and 2 mM L-glutamine. For B cell culture, anti-CD40 (20 ng/ml) was added to the culture to avoid apoptosis throughout the study. The

viability was greater than 98% before using for further experiments as checked by Trypan blue exclusion assay.

Flow cytometry

Cells were stained with fluorochrome-labeled antibodies (or isotype IgG) at 0.5 μg/ml for 1 h at room temperature. The expression of surface markers was assessed with a flow cytometer (FACSCanto I, BD Biosciences). In the case of intracellular staining, the cells (fresh or stained for the surface markers) were fixed with 1% paraformaldehyde mixed with 0.5% saponin for 1 h. After washing, the cells were stained with fluorochrome-labeled antibodies (or isotype IgG) for 1 h at room temperature and then analyzed with a flow cytometer. The data were analyzed with the software flowjo. The data of isotype IgG staining were used as a reference to set the gates. In the case of cell proliferation assay, the cells of interest were labeled with CFSE (Carboxyfluorescein succinimidyl ester) and cultured with appropriate procedures, and analyzed by the CFSE-dilution assay.

Assessment of apoptosis

After appropriate treatment, the cells were collected from the culture, stained with Annextin V reagent and propidium iodide (PI), and analyzed by flow cytometry. The Annexin V positive cells, or both Annexin V/PI positive cells were regarded as apoptotic cells.

Real time quantitative RT-PCR (RT-qPCR)

The total RNA was extracted from the cells of interest. A cDNA was synthesized with the RNA using a reverse transcription kit. The qPCR was performed on a real time PCR device (MiniOpticon, Bio-Rad) with the SYBR Green Master Mix. The results were calculated with the $2^{-\Delta\Delta Ct}$ method. The results of mRNA are presented as relevant changes against controls. The primers using in this study include: IgE, tagtgactctgatgccaccc and ccccagaggtccaagtaaca. IL-10, gttctttggggagccaacag and gctccctggtttctcttcct. β-actin, cgcaaagacctgtatgccaa and cacacagagtacttgcgctc.

Preparation of cytosolic and nuclear extracts

Cells were incubated with lysis buffer (10 mM HEPES, pH 7.4, 10 mM NaCl, 1.5 mM MgCl2, 0.5 mM DTT, 0.2% Nonidet P-40, and 0.2 mM PMSF) at 4°C for 15 min, and centrifuged at 500 ×g for 10 min at 4°C. The supernatant was collected as the cytosolic extract. The pellet was added with nuclear extract buffer (20 mM HEPES-KOH, pH 7.9, 25% glycerol, 420 mM NaCl, 1.5 mM MgCl2, 0.2 mM EDTA, 0.5 mM DTT, 0.2 mM PMSF, and 1× protease inhibitor cocktail) and incubated for 15 min at 4°C, followed by centrifugation at 13,000 ×g for 10 min at 4°C. The supernatant was collected as the nuclear extract. The protein concentrations were determined by the Bradford method.

Western blotting

Proteins were extracted from the cells. The proteins were fractioned with sodium dodecyl sulfate polyacrylamide gel electrophores (SDS-PAGE) and transferred onto a PVDF membrane.

After blocking with 5% skim milk for 30 min, the membrane was incubated with the primary antibodies at 300 ng/ml overnight at 4°C and followed by incubating with the second antibodies (labeled with peroxidase) for 1 h at room temperature. Washing with TBST (Tris-buffered saline Tween 20) was performed after incubation. The membrane was developed with enhanced chemiluminescence (ECL). The results were photographed with an imaging device (UVI system; Beijing, China).

Validation of commercial DME (DME#)

To validate the commercial DME, ELISA was performed with DME# or DME (prepared from dust mites). The immune response of DME and DME# is presented below (Fig. S2).

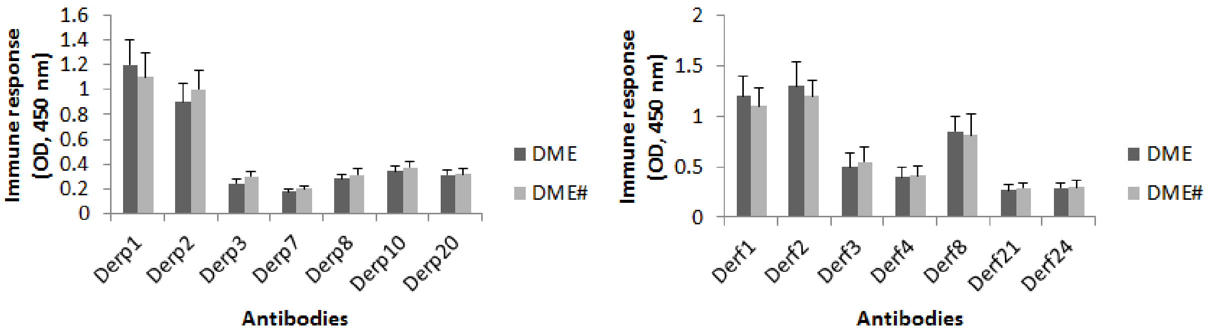

Figure S1. Immune response of dust mite extracts (DME). DME was prepared with dust mite by us. DME# is commercial DME, which was used in SIT of the present study. The 96 well plates were coated with 100 µl of DME or DME# at a concentration of 1 µg/mL in carbonate buffer and stored at 4°C overnight with 3% BSA. The plates were incubated with monoclonal antibodies of subtypes of mite allergens that were prepared by us as denoted on the X axis at 37 °C for 2 h. Then the plates were added with 100 µl of HRP labeled anti-mouse antibody (1:2000 v/v) and incubated at 37°C for 2 h. The plates were subjected to color development

with TMB at 37°C for 10 minutes, and the reactions were terminated by adding 2 mol/L H_2SO_4. The plates were read with a microplate reader at 450 nm. The bars indicate the immune response of DME and DME#. Data of bars are presented as mean ± SD. There is no significant difference in the immune response between DME and DME#. The data were summarized from 3 independent experiments.

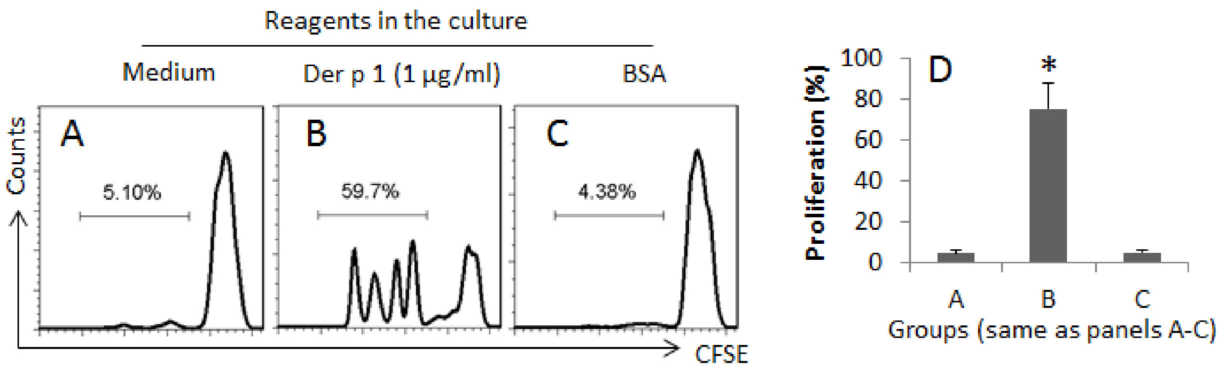

Figure S2. Assessment of peripheral Derp1-specific B cells. A-C, the flow cytometry histograms indicate the frequency of Derp1-specific B cells in the PBMCs from AR patients sensitized to dust mites. The patient number and conditions are presented in figure 1. D, the bars indicate the summarized data of A-C. The data are presented as mean ± SD. *, p<0.01, compared with the group A.

Table S1. Specific B cell (sBC) number

Patient#	PBMC	BC	sBCbefore	sBCafter
**1**	100000	17451	7079	16129
**2**	100000	19874	8415	18742
**3**	100000	18120	8280	14524
**4**	100000	17852	7541	16412
**5**	100000	18120	7007	13641
**6**	100000	17856	7485	15891
**7**	100000	17882	6855	17539
**8**	100000	18432	7674	19773
**9**	100000	18741	7603	19208
**10**	100000	17725	6689	14629
**11**	100000	18863	7985	20008
**12**	100000	17952	6858	17598
**Mean**		18241.83	7345.75	
**SD**		658.13	524.81	
**p**			4.03E-23	

Before: Before proliferation.

After: After proliferation.
